# Harnessing synthetic biology for sustainable biomining with Fe/S-oxidizing microbes

**DOI:** 10.3389/fbioe.2022.920639

**Published:** 2022-09-05

**Authors:** Jinjin Chen, Yilan Liu, Patrick Diep, Radhakrishnan Mahadevan

**Affiliations:** ^1^ Department of Chemical Engineering and Applied Chemistry, University of Toronto, Toronto, ON, Canada; ^2^ Institute of Biomedical Engineering, University of Toronto, Toronto, ON, Canada

**Keywords:** synthetic biology, Fe/S-oxidizing microbes, biomining, design-build-test-learn (DBTL) cycle, CRISPR

## Abstract

Biomining is a biotechnological approach where microorganisms are used to recover metals from ores and waste materials. While biomining applications are motivated by critical issues related to the climate crisis (*e.g.*, habitat destruction due to mine effluent pollution, metal supply chains, increasing demands for cleantech-critical metals), its drawbacks hinder its widespread commercial applications: lengthy processing times, low recovery, and metal selectivity. Advances in synthetic biology provide an opportunity to engineer iron/sulfur-oxidizing microbes to address these limitations. In this forum, we review recent progress in synthetic biology-enhanced biomining with iron/sulfur-oxidizing microbes and delineate future research avenues.

## Introduction

Biomining is a technique that utilizes microorganisms to recover target metals from ores and waste materials, and potentially capture them for recovery and downstream purification. Chemolithotrophic iron/sulfur (Fe/S)-oxidizing microbes have attracted attention in biomining research since they thrive in extremely acidic metal-rich environments, produce a bioleaching lixiviant containing Fe^3+^ and H^+^ by oxidizing ferrous iron and/or reduced inorganic sulfur compounds (RISCs) as an energy source, and fix CO_2_ from the atmosphere as the carbon source (Chen et al., 2022a). Considerable efforts have been devoted to applying synthetic biology to improve the biomining performance of Fe/S-oxidizing microbes, and significant progress has been made in recent years. This mini review presents the up-to-date developments of synthetic biology in biomining with Fe/S-oxidizing microbes and provides perspectives on this research area.

## Fe/S-oxidizing microbes in biomining processes

**FIGURE 1:**
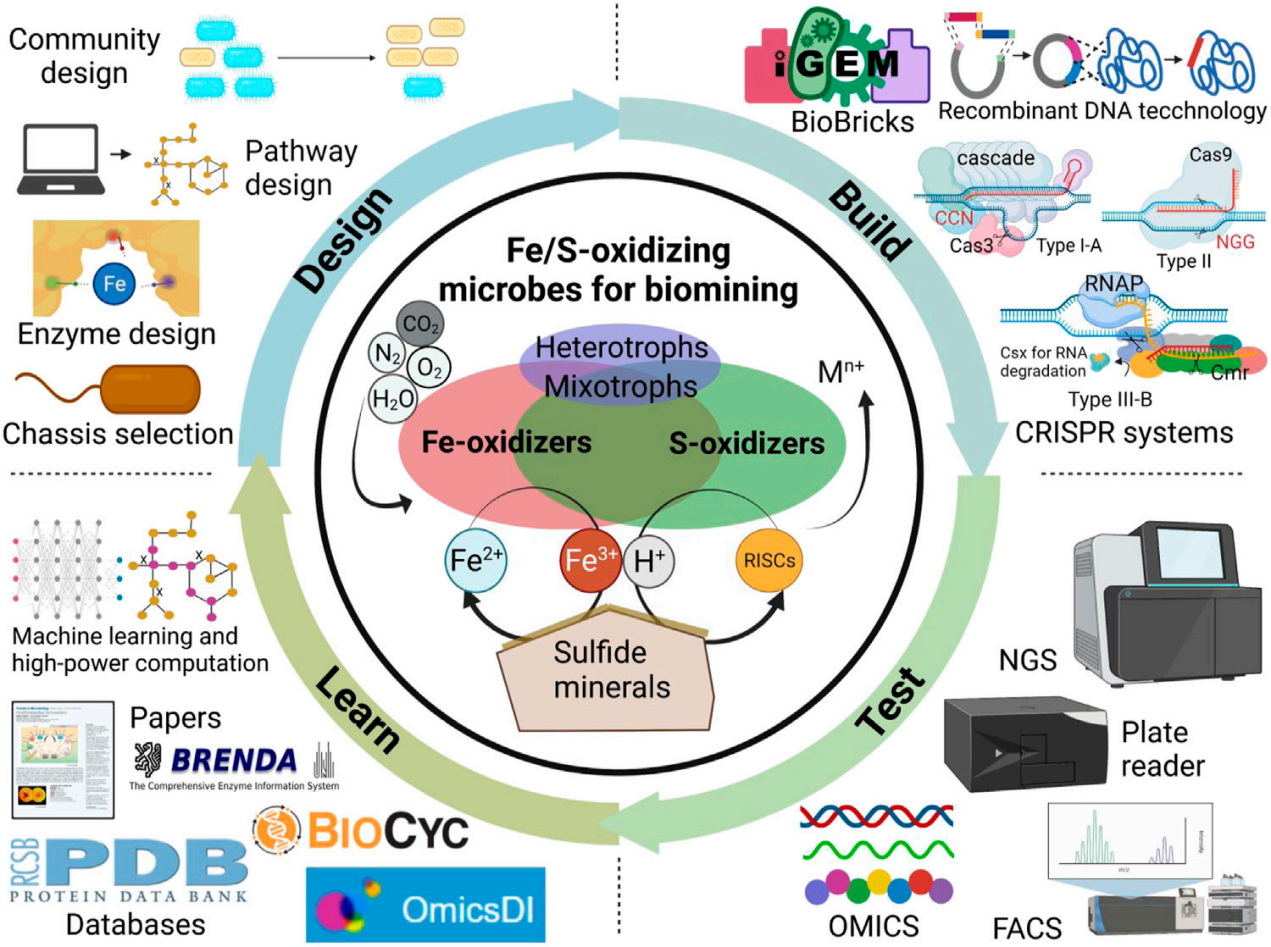
Harnessing synthetic biology for sustainable biomining with Fe/S-oxidizing microbes. Technology advances accelerate the design-build-test-learn cycle of synthetic biology-assisted biomining. NGS, next-generation sequencing; FACS, fluorescence-activated cell sorting, OMICS refers to genomics, proteomics, transcriptomics, metabolomics, and fluxomics.

A typical biomining microbial community consists of autotrophic Fe/S-oxidizers, mixotrophs and heterotrophs (Li and Wen, 2021) (Figure 1). Neutrophilic Fe-oxidizers (eg. *Rhizobiales* spp. and *Ralstonia* spp.) may play a role to kick start the mineral oxidation at circumneutral pH (Percak-Dennett et al., 2017). The acidophilic Fe-oxidizers (*e.g.*, *Leptospirillum* spp. and *Ferroplasma* spp.) obtain electrons from dissolved Fe^2+^ for energy generation, thus generating Fe^3+^ that can attack the mineral’s lattice structure to release additional iron, target metals and sulfur compounds. However, S^0^ or jarosite (KFe_3_(SO_4_)_2_(OH)_6_) can accumulate to form a passivation layer covering the mineral surface thus inhibiting further leaching. S-oxidizers (*e.g.*, *Acidianus* spp., some species in *Acidithiobacillus* and *Sulfolobus*) can oxidize sulfur compounds like S^0^ to sulfuric acid. This process generates additional H^+^ that assists Fe^3+^ in further degrading the mineral lattice structure to release the target metal. Combined, Fe-oxidizers and S-oxidizers maintain extremely acidic conditions with a high oxidation-redox potential (ORP) to leach target metals from the mineral without precipitating them. Moreover, mixotrophs and heterotrophs found in genera such as *Acidiphilium, Metallosphaera, Sulfobacillus* and *Sulfolobus* can also degrade organic compounds mitigating potential toxicity issues to autotrophic Fe/S-oxidizers.

Several species in the genera *Acidithiobacillus* are capable of oxidizing both Fe^2+^ and RISCs. In particular, *Acidithiobacillus ferrooxidans* is the most widely studied chassis for biomining applications. Mixed cultures of *A. ferrooxidans* with other Fe-oxidizing and S-oxidizing microbes, mixotrophs and heterotrophs have been shown to be effective for bioleaching (Wang et al., 2020). Hence, *A. ferrooxidans* with its metabolic versatility and ability to fix CO_2_ is a prime target for synthetic biology efforts to enhance biomining kinetics, to produce value-added products from CO_2_ and to confer advanced functionality like selective metal recovery. In addition, new Fe/S-oxidizing microbes found in extreme environments will also contribute valuable genetic diversity, such as the novel archaeal group found in Yellowstone National Park and *Zetaproteobacteria* from marine hydrothermal vents (Kozubal et al., 2013; McAllister et al., 2019).

## Design-build-test-learn applied to engineering Fe/S-oxidizing microbes

A hallmark of synthetic biology is the application of principles outlined in the design-build-test-learn (DBTL) cycle commonly found in traditional engineering disciplines to the field of biology. This DBTL cycle integrates rational design, genetic engineering, results testing, and data analysis to learn and resolve bottlenecks in biomanufacturing. Such a DBTL cycle could be specifically applied to engineering Fe/S-oxidizing microbes for biomining applications (Figure 1). For *Design*, computational modelling tools combined with biological databases (e.g., genome sequences, OMICS datasets) enable the rational design of enzymes, metabolic pathways, and communities of biomining microbes. A genome-scale metabolic model (GEM) of *A. ferrooxidans* (iMC507) is available for *in silico* analysis and design, and has been used to predict gene deletions for growth-coupled extracellular polymeric substance (EPS) production (Campodonico et al., 2016).

For *Build*, there are genetic engineering tools to engineer Fe/S-oxidizing microbes with desirable biomining features. Some conjugative/mobilizable plasmids and viruses have been reported in species of *Acidianus*, *Acidiphilium*, *Leptospirillum*, *Metallosphaera* and *Sulfobacillus* to allow for heterologous gene expression (Beard et al., 2021). However, significant advances have been made in *Acidithiobacillus* and archaea *Sulfolobus* since prior efforts were mostly devoted to studying these model organisms for acidophiles. For *Sulfolobus* spp., the endogenous type I-A and type III-B CRISPR/Cas systems have been harnessed for efficient targeted genome editing and gene silencing (Peng et al., 2017). Comparatively, genetic engineering of *Acidithiobacillus* spp. is time-consuming due to its generally slower growth rate, thus making it harder to engineer. Nonetheless, studies have shown it is still possible to use conventional genome editing methods like transposon-based gene interruption or suicide plasmid-based gene deletion (Chen et al., 2022a). Recently, Moya-Beltrán *et al.* investigated type IV CRISPR-Cas systems in the class *Acidithiobacillia*, which may enable genome editing like *Sulfolobus* spp. (Moya-Beltrán et al., 2021). Recently, there was a report of a successful gene knockdown in *A. ferrooxidans* using the CRISPR-dCas9 system derived from *S. pyogenes* (Yamada et al., 2022). At the same time, our group first achieved seamless genome editing using the CRISPR-Cas9 system in *Acidithiobacillus ferridurans*, which significantly accelerates the genetic engineering process (Chen et al., 2022b). However, additional studies are needed to apply CRISPR systems in *Acidithiobacillus* strains and other Fe/S-oxidizing microbes for biomining applications.

The *Test* and *Learn* phases of the DBTL involve validating and analyzing data generated from engineered strains for subsequent engineering. These components of the DBTL cycle are most underdeveloped for biomining microbes. The development of high-throughput technologies such as next-generation sequencing (NGS), automated liquid handling/plate reader workflows, fluorescence-activated cell sorting (FACS), and microfluidics have facilitated the screening of large strain libraries (Sarnaik et al., 2020). These big datasets combined with existing databases like OmicsDI, BioCyc, BRENDA, and PDB can enable machine learning methods to generate critical insights to inform design iterations. While for example high-throughput sequencing of 16S rRNA gene has been used in biomining studies to monitor microbial community dynamics, efforts are still needed in high-throughput engineering, culturing, testing, screening of Fe/S-oxidizing microbes with desirable biomining traits.

## Advances of synthetic biology-enhanced biomining

While a comprehensive DBTL methodology is yet to be applied for engineering biomining, there are several examples where synthetic biology has been deployed in this context. Gumulya et al. (2018) reviewed the manipulation of genes responsible for acid tolerance, metal tolerance, osmotolerance, thermotolerance, Fe/S-oxidation, and carbon fixation to improve microbe’s suitability for industrial use. In *Acidithiobacillus* strains, overexpression of *rus* and *cyc2* genes was found to improve Fe^2+^ oxidation activity, whereas overexpression of the quorum sensing (QS) operon *afeI*-*orf*-*afeR* improved sulfur oxidation rates and promoted EPS synthesis. Glutathione biosynthesis gene *gshB* overexpression significantly increased intracellular reactive oxygen species (ROS) levels and expanded the halotolerance of the cells. Other genes related to Fe/S-metabolisms (e.g., *fur*, *tetH*, *sdo1, sdo2, rsrR, rsrS,* and *sor*), metal resistance (e.g., *cueO*, *ars* and *mer*), c-di-GMP signaling (e.g., *dgc* and *pelD*) and quorum sensing (e.g., *aar*, *ado* and *act*) were also investigated (Jung et al., 2021).

A deeper understanding of Fe/S-oxidizing microbes’ genes is nonetheless still needed for more detailed metabolic engineering towards desirable biomining properties, compared to the model chassis *Escherichia coli* and *Saccharomyces cerevisiae*. Schmitz et al. (2021) harnessed high-throughput genome editing and sequencing approaches in the study of heterotrophic bacterium *Gluconobacter oxydans* to produce organic acids for rare earth elements biomining. They created a library of single-gene transposon mutants in *G. oxydans* and found the bioleaching rates of rare earth elements increased up to 18% when phosphate-specific transport systems genes were disrupted. Therefore, high-throughput engineering, culture, sequencing, screening processes may facilitate faster development of engineered Fe/S-oxidizing microbes.

## The outlook of synthetic biology-enhanced biomining applications

Biomining processes are commercially applied around the world in two broad categories: irrigation-type and stirred tank-type. Irrigation-type examples include heap bioleaching processes carried out by companies like Newmont Mining and Phelps Dodge, which are suitable for metal recovery from low-grade ores because these systems are easier and cheaper to construct, although the process can be slow with inefficient recoveries (Petersen, 2016). In contrast, the more widely used processes are of stirred tank-type, such as BIOX™ technology from Metso-Outotec. The control of operating parameters contribute significantly to the fast and efficient metal recovery, but the operating cost is higher (Rawlings et al., 2003). Recently, Kremser et al. (2020) compared the potential of heap and stirred-tank bioreactors for metal recovery from shredder-light-fractions. Pure and co-culture of *A. ferrooxidans* and *L. ferrooxidans* were used for Cu, Zn and Ni recovery in batch and up-scale experiments, which highlights the potential for future commercial biomining applications with engineered Fe/S-oxidizing microbes. However, for large-scale applications, both heap and stirred-tank biomining provide an open, non-sterile environment. This open environment can lead to difficulties in optimizing a stable microbial consortia that can outcompete the native consortia and match the scale and complex nature of the ore feedstocks (Rawlings and Johnson, 2007). The design and construction of synthetic and mixed microbial consortia is an emerging area in synthetic biology, and may help to address these issues for better industrial biomining applications (Brune and Bayer, 2012; McCarty and Ledesma-Amaro, 2019).

## Concluding remarks and future perspectives

Synthetic biology-enhanced biomining can be a sustainable, eco-friendly, and cost-effective technology with applications in leaching and recovering base metals, precious metals, and rare earth elements from a variety of feedstocks like low-grade ores, mine wastes, and electronic wastes. Though engineering Fe/S-oxidizing microbes is still at its earliest stage, we believe that the following developments can accelerate synthetic biology-enhanced biomining research:

(1) Fast and efficient genome editing methods and tools are needed for Fe/S-oxidizing microbes (i.e., CRISPR/Cas9 system). Genetic tools are available for *Sulfolobus* strains that are adapted to high temperature (80^o^C) and acidic conditions (pH < 3), highlighting their suitability for engineering (Lewis et al., 2021). We summarized the diverse genetic tools that are feasible for *Acidithiobacillus* species (Chen et al., 2022a). Different CRISPR systems have been successfully used for genome editing in *Sulfolobus* and *Acidithiobacillus* species. We believe genome editing methods for other Fe/S-oxidizing microbes can offer us a larger solution space for designing synthetic biomining consortia with desirable leaching properties.
(2) Development of high-throughput technologies to culture and screen of Fe/S-oxidizing microbes will accelerate the engineering of strains with more desirable industrial properties such as heavy metal tolerance, robustness under large bioreactor conditions. Example studies could include designing synthetic histidine kinases that can sense heavy-metals (Sarnaik et al., 2020), creating a whole-genome single-gene mutant library to enable screens for desired properties (Schmitz et al., 2021), or using CRISPRi system as an efficient platform for rapid identification of target genes (Wu et al., 2020).
(3) The leaching of metals from the mineral body is typically not a selective process as all metals contained in the mineral lattice are released into solution. Such a complex leachate complicates downstream processing. To mitigate this complexity, metal-sensing regulators (e.g., riboswitches) could be used to construct circuits that enable selective and sequential metal recovery by Fe/S-oxidizing microbes by regulating pathways responsible for specific metal uptake, storage, and efflux during biomining process (Diep et al., 2018).
(4) Engineering microbial communities using synthetic biology tools, such as CRISPR/Cas systems, riboswitches, quorum sensing or syntrophic exchanges (McCarty and Ledesma-Amaro, 2019) may promote synergistic effects among Fe-oxidizers, S-oxidizers and heterotrophs and further enhance biomining efficiency, especially in heap and stirred-tank biomining that carried out in the non-sterile environment.
(5) Rather than directly genetically engineering Fe/S-oxidizing bacteria, directed evolution may also be a good research direction since stakeholders are more likely to accept industrial bacteria with more natural origins. For example, the EvolvR system can help evolve industrial microbes with desired phenotypes, such as fast growth rate, high tolerance to complex metals, acidity, salinity, and more (Halperin et al., 2018).
